# Gut microbiota: a newly identified environmental factor in systemic lupus erythematosus

**DOI:** 10.3389/fimmu.2023.1202850

**Published:** 2023-07-18

**Authors:** Kaijin Yao, Yina Xie, Jiali Wang, Yongda Lin, Xiutian Chen, Tianbiao Zhou

**Affiliations:** Department of Nephrology, the Second Affiliated Hospital of Shantou University Medical College, Shantou, China

**Keywords:** systemic lupus erythematosus, gut microbiota, dysbiosis, immune dysregulation, cytokines

## Abstract

Systemic lupus erythematosus (SLE) is a chronic autoimmune disease that predominantly affects women of childbearing age and is characterized by the damage to multiple target organs. The pathogenesis of SLE is complex, and its etiology mainly involves genetic and environmental factors. At present, there is still a lack of effective means to cure SLE. In recent years, growing evidence has shown that gut microbiota, as an environmental factor, triggers autoimmunity through potential mechanisms including translocation and molecular mimicry, leads to immune dysregulation, and contributes to the development of SLE. Dietary intervention, drug therapy, probiotics supplement, fecal microbiome transplantation and other ways to modulate gut microbiota appear to be a potential treatment for SLE. In this review, the dysbiosis of gut microbiota in SLE, potential mechanisms linking gut microbiota and SLE, and immune dysregulation associated with gut microbiota in SLE are summarized.

## Introduction

Systemic lupus erythematosus (SLE), mainly affecting women of childbearing age, is a chronic autoimmune disease that involves multiple organs such as the skin, joints, kidneys, and central nervous system (1). The estimated global incidence and prevalence of SLE were 5.14 per 100,000 person-years (1.4 to 15.13) and 43.7 per 100,000 person-years (15.87 to 108.92), respectively (2).

SLE is characterized by immune dysregulation and loss of tolerance to autoantigens (3). Both innate and adaptive immunity are involved in the pathogenesis of SLE (4). Autoreactive B cells which are induced by the breakdown of central and peripheral tolerance mechanisms, occupying a core position in the adaptive immune response of SLE, produce autoantibodies, present autoantigens and activate autoreactive T cells (4, 5). CD4^+^T helper cell subsets such as Th1, Th2, Th17, T follicular helper and regulatory T cells exist disturbances in number and function. The reduced cytolytic activity of CD8^+^ cytotoxic T cells induces higher rates of infection and sustenance of autoimmunity (6). The population of clusters of double-negative T cells CD4^-^CD8^-^ T cells, which can produce inflammatory cytokines and infiltrate target tissues, is increased in SLE patients (7, 8). In the innate immune response, abnormal activation or disabled tolerance of dendritic cells induces abnormal production of type I interferons and inflammatory mediators, which contributes to pathogenic innate immunity and autoinflammation (9). In SLE, the defective clearance of apoptotic material and neutrophil extracellular traps lead to the exposure of autoantigens and trigger the production of autoantibodies (10, 11). Local depositions of the immune complexes that are generated between autoantibodies and autoantigens result in serious inflammation through activating the complement system (12, 13).

The etiology of SLE is still not entirely clear. It is now believed that genetic and environmental factors mainly contribute to the development of SLE (14). The genome-wide association study has identified more than 60 risk loci for SLE susceptibility (15). Individuals with identified genetic polymorphisms have a higher risk of developing SLE than the general population (16). Environmental factors including ultraviolet light (17), silica exposure (18), cigarette smoking (19), viral and bacterial infections (20), and sex hormones (21) are involved in the pathogenesis of SLE. Currently, the main therapeutic drugs for SLE are glucocorticoids, immunosuppressive drugs and antimalarial drugs, but their use is limited due to serious side effects (22, 23). Gut microbiota connects some external environmental effectors with the immune system and supports the immune system program to tolerate innocent external and self-antigens (24). However, when the gut microbiota is dysbiotic, it can disrupt immune function, induce inflammation and immune system sensitization and lead to autoimmune diseases (25, 26). Recently, increasing studies have found that gut microbiota as an environmental factor contributes to the development of SLE (27), and modulating the gut microbiota appears to be a potential treatment for SLE (28). In this review, we summarize the alteration of gut microbiota in SLE, potential mechanisms connecting gut microbiota to SLE, and immune dysregulation related to gut microbiota in SLE.

## The alteration of gut microbiota in SLE

### The changes of gut microbiota in lupus mice models

Recently, increasing studies have revealed that dysbiosis of gut microbiota is associated with SLE. Alteration of the gut microbiota has been found in various lupus mouse models, as shown in **Table 1**. Luo et al. (31) found that the gut microbiota changed significantly before and after lupus onset in NZB/W F1 mice. Zhang et al. (29) found that *Lactobacillaceae* significantly decreased and *Lachnospiraceae* significantly increased in lupus-prone MRL/lpr mice. The decreased abundance of *Lactobacillaceae* was also observed in MRL/lpr mice in another study (36). Increased intestinal abundance of *Lactobacillaceae* was associated with improvement of lupus symptoms, while increased colonization of *Lachnospiraceae* was associated with disease progression (29). Consistently, Mu et al. (30) found that *Lactobacillales* significantly depleted in the gut microbiota of MRL/lpr mice and was associated with improvement of lupus symptoms. However, in another lupus mice model NZB/W F1 mice, Luo et al. (31) reported that the greater abundance of a group of *Lactobacilli* was linked with more severe clinical signs. Zegarra-Ruiz et al. (32) found that the abundance of *Lactobacill. reuteri* increased in TLR7.1 Tg mice and *Lactobacill. reuteri* colonization exacerbated systemic autoimmunity under specific-pathogen-free and gnotobiotic conditions. In addition, He et al. (34) observed a rise in the abundance of *Bacteroidetes* and a reduction in *Firmicutes* in the gut microbiota of MRL/lpr mice. Abundant *Bacteroidetes* and decreased *Firmicutes* were also found in NZBWF1 mice, and increased proportions of *Bacteroides* were associated with high blood pressure (35). Abdelhamid et al. (33) found that the abundance of *Bacteroidetes* was positively correlated with glomerular pathological scores. A lower *Firmicutes*/*Bacteroidetes*(F/B) ratio found in 6-week-old MRL/lpr mice might play an important role in promoting early disease onset (38). Furthermore, Chen et al. (39) reported that the increased abundance of genera *Candidatus saccharimonas*, *Desulfovibrio*, *Odoribacter* and *Roseburia* in mice treated with HCMVpp65 peptide is significantly correlated with lupus-like effects including enhanced levels of creatinine, proteinuria, glomerular damage and anti-dsDNA antibodies. Valiente et al. (40) found that NZM2410 mice colonized with segmented filamentous bacteria showed worsening glomerulonephritis, deposition of glomerular and tubular immune complexes, and interstitial inflammation. Therefore, gut microbiota dysbiosis in lupus mice models could be characterized by a decrease of beneficial bacteria and an increase of harmful bacteria and was associated with SLE.

**Table 1:** The alteration of gut microbiota in lupus mice models.

Study(Year)	Mice	The alteration of gut microbiota	Association	Intervention outcome	Reference
Zhang, et al. (2014)	MRL/lpr mice	**Family:** Lactobacillaceae ↓, Lachnospiraceae ↑, Ruminococcaceae ↑, Rikenellaceae (genus Alistipes)↑	Lactobacillaceae was negatively correlated with lupus activity. Lachnospiraceae was associated with more severe lupus symptoms.	Retinoic acid treatment restored lactobacilli and improved lupus symptoms.	(29)
	B6/lpr mice	**Family:** Clostridiaceae ↑, Lachnospiraceae ↑			
Mu, et al. (2017)	MRL/lpr mice	**Family:** Lactobacillaceae ↓	Lactobacillales was associated with the improvement of lupus symptoms.	Lactobacillus treatment may correct the leakiness of gut and attenuate lupus nephritis by limiting renal deposition of IgG2a and restoring Treg/Th17 balance.	(30)
Luo, et al. (2018)	NZB/W F1 mice	Several species in the genera Clostridium, Dehalobacterium, Lactobacillus, Oscillospira, Dorea, Bilophila, and AF12 and an unnamed genus within the family Ruminococcaceae ↑, Akkermansia muciniphila and a species within the genus Anaerostipes ↓	The greater abundance of a group of lactobacilli was associated with more severe clinical signs.		(31)
Zegarra-Ruiz, et al. (2019)	TLR7.1 Tg mice	**Family:** Rikenellaceae ↑ **Genus:** Desulfovibrio ↑ **Species:** Lactobacill. reuteri ↑	Lactobacill. reuteri alone was sufficient to exacerbate systemic autoimmunity.	Resistant starch decreased the abundance of L. reuteri, gut leakiness, type I IFN and proinflammatory responses, pDC infiltrations, and organ pathology, thereby preventing the development of systemic autoimmunity.	(32)
Abdelhamid, et al. (2020)	Balb/c mice treated with pristane	**Order:** Bacterodiales ↑ **Genus:** Lactobacillus ↓, Ruminococcus ↑ **Species:** Lactobacillus gasseri ↓	The abundance of Bacteroidetes was positively correlated with glomerular pathological scores.	All-trans-retinoic acid treatment decreased circulatory and renal deposition of autoantibodies as well as suppressed the renal expression of proinflammatory cytokines and chemokines.	(33)
He, et al. (2020)	MRL/lpr mice	**Phyla:** Firmicutes ↓, Bacteroidetesma ↑ **Class:** Clostridia ↓, Bacteroidia ↑ **Order:** Clostridiales ↓, Bacteroidales ↑ **Family:** Lachnospiraceae ↓		Butyrate supplementation can ameliorate gut microbiota dysbiosis and reduce kidney damage.	(34)
de la Visitación, et al. (2021)	NZBWF1 mice	**Phyla:** Verrucomicrobia, Proteobacteria, Bacteroidetes and Proteobacteria ↑, Firmicutes ↓ **Genus:** Parabacteroides, Pedobacter, Olivibacter and Clostridium ↑	Increased proportions of Bacteroides were linked with high blood pressure.	Antibiotic treatments induced changes in gut microbiota, and inhibited the increment of blood pressure, target organ hypertrophy, renal injury and disease activity.	(35)
Kim, et al. (2021)	MRL/lpr mice	**Family:** Lactobacillaceae ↓ **Genus:** Lactobacillus ↓		Lactobacillus acidophilus improved gut dysbiosis and decreased the renal inflammation.	(36)
Toumi, et al. (2022)	BALB/cByJ mice treated with pristane	**Phyla:** Tenericutes ↓ **Family:** Tannerellacea ↑ **Genus:** Parabacteroides ↑, Bacteroides ↑, Alistipes ↑			(37)

↑, higher abundance or enriched; ↓, lower abundance or depleted.

The gut microbiota from lupus patients and mice in recipient mice can cause the production of autoantibodies and increase the expression of lupus-related genes. Ma et al. (41) conducted a study transplanting fecal microbiota from SLE mice to germ free mice and found that fecal microbiome from SLE mice could induce the production of anti-dsDNA antibodies and upregulate the expression of SLE susceptibility genes in germ free mice. Choi et al. (42) transferred the dysbiotic gut microbiota from triple congenic lupus-prone mice into germfree congenic C57BL/6 mice, and found that the gut microbiota activated immune cells and induced the production of autoantibodies in recipient mice. In a study by Ma et al., when receiving fecal microbiota from SLE patients, germ-free mice showed increased expression of genes associated with SLE and a range of lupus-like phenotypic features, including imbalanced cytokines, elevated serum levels of autoimmune antibodies, changed the distribution of immune cells in mucosal and peripheral immune responses (43). In addition, high blood pressure and vascular complications were found in germ-free or germ-depleted mice after receiving gut microbiota from hypertensive NZBWF1 mice (35). These results further illustrate that dysbiosis of gut microbiota contributes to the development of SLE.

### The changes of gut microbiota in SLE patients

**Figure 1:**
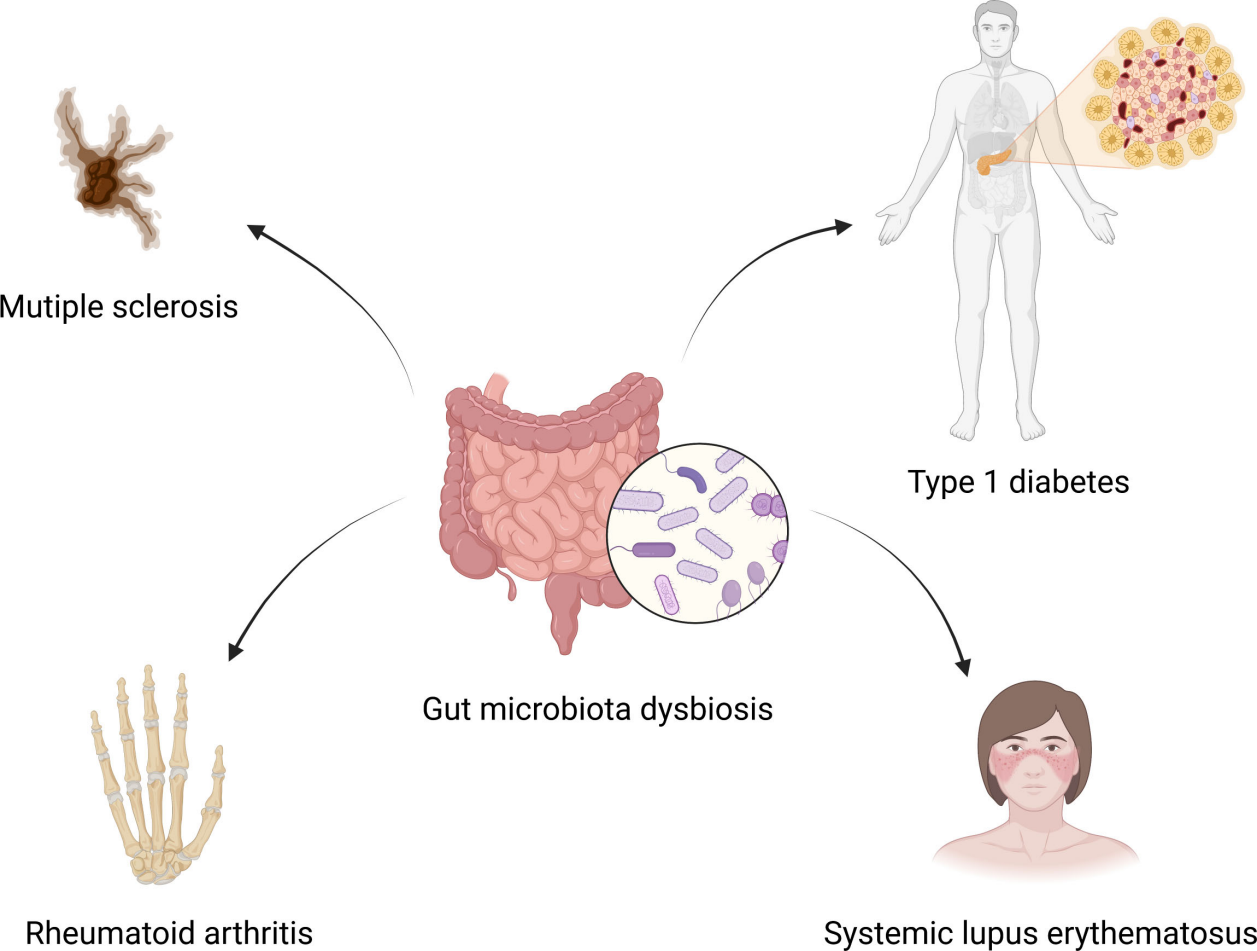
Gut microbiota dysbiosis and extra-intestinal autoimmune diseases. Gut microbiota dysbiosis is associated with multiple sclerosis, rheumatoid arthritis, type 1 diabetes and systemic lupus erythematosus. Created by BioRender.com.

It has been reported that patients with extra-intestinal autoimmune diseases, including multiple sclerosis (44), rheumatoid arthritis (45), and type 1 diabetes (46), have distinct gut microbiota compositions compared to healthy individuals. Plenty of studies have also shown that gut microbiota altered significantly in SLE patients compared to healthy controls. Extra-intestinal autoimmune diseases associated with gut microbiota dysbiosis are summarized in **Figure 1**. Hevia et al. (47) performed a study comparing the fecal microbial profiles between SLE patients and healthy subjects and published the first report to describe an SLE-associated intestinal dysbiosis in humans. Wang et al. (48) compared lupus patients to their healthy family members, controlling for living conditions and dietary factors, and found that the gut microbiota of lupus patients still differed from that of healthy controls. It is known that *Bacteroidetes* and *Firmicutes* are the most abundant components of the human gut microbiota (49). A significantly lower F/B ratio was observed in SLE patients compared to healthy subjects (47). Several subsequent studies conducted in different countries around the world have also observed a lower F/B ratio in the gut microbiota of lupus patients compared to healthy people (37, 50–52). Furthermore, Katz-Agranov and Zandman-Goddard (53) revealed that SLE individuals even in remission had a significantly lower F/B ratio. Active SLE patients showed a significantly lower F/B ratio compared to the inactive SLE group (37). In addition, Gerges et al. (52) found that the F/B ratio was negatively correlated with the SLEDAI-2K score. Widhani et al. (54) also reported that SLE patients with mild disease activity had a higher F/B ratio compared to patients with moderate or high disease activity. These results indicate that the lower F/B ratio in SLE patients is independent of ethnicity, lifestyle as well as disease stage and inversely correlated with lupus disease activity. However, a lower F/B ratio is not the specific feature in the alteration of gut microbiota in SLE patients, and it is also linked to other diseases, such as type 2 diabetes (55), Crohn’s disease (56) and Parkinson’s disease (57). Reduced bacterial diversity is another main feature of intestinal dysbiosis that has been reported by many studies conducted in humans (31, 51, 58–60). Furthermore, patients with a high SLE activity index had a particularly significant reduction in the species diversity of gut microbiota (58). In addition, a recent study reported that the gut microbiota composition of SLE patients with depression was also different from that of SLE patients without depression (61).

The indicators commonly used to reflect the disease activity of SLE patients include SLEDAI, anti-double stranded DNA (anti-dsDNA), erythrocyte sedimentation rate (ESR), C-reactive protein (CRP) and complement C3 (62–64). Some biomarkers in the gut microbiota of SLE patients have been found to be associated with the disease activity indicators. Bagavant et al. (65) found that higher titers of anti-*E. gallinarum* IgG in patients were significantly correlated with the presence of anti-Ribosomal P, anti-dsDNA and anti-Sm autoantibodies. Azzouz et al. (58) reported that *Ruminococcus gnavus* (RG) showed a mean 5-fold overabundance in lupus patients compared with healthy controls. And patients with high disease activity and especially lupus nephritis showed the greatest expansion of RG. Furthermore, anti-RG antibodies were directly correlated with anti-DNA levels and SLEDAI scores, while they were negatively correlated with C3 and C4. Patients with active nephritis including Class III and IV exhibited the highest levels of anti-RG strain restricted antibodies in serum. The abundance of *Acholeplasma*, *Capnocytophaga* and *Leptotrichia* were negatively correlated with SLEDAI score, and the abundance of *Bacteroides*, *Ruminococcus* and *Akkermansia* had an inverse correlation with the serum levels of complement C3 (60). The genus *Streptococcus* was associated with the lupus activity (59). These findings suggest that some biomarkers in the gut microbiota are linked with the disease activity of SLE patients and have the potential to be used as indicators to reflect the disease activity of SLE patients. Studies on the gut microbiota in lupus patients are summarized in **Table 2**.

**Table 2:** The alteration of gut microbiota in SLE patients.

Study(Year)	Human Subjects(n)	Region	The alteration of gut microbiota	Association	Reference
Hevia, et al. (2014)	SLE (20) vs. HC (20)	Spain	**Phyla:** Firmicutes/Bacteroidetes ↓, Bacteroidetes ↑		(47)
He, et al. (2016)	SLE (45) vs. HC (48)	China	**Phyla:** Bacteroidetes, Actinobacteria and Proteobacteria ↑, Firmicutes ↓ **Genus:** Rhodococcus, Eggerthella, Klebsiella, Prevotella, Eubacterium, Flavonifractor and Incertae sedis ↑, Dialister and Pseudobutyrivibrio ↓		(50)
Luo, et al. (2018)	SLE (14) vs. HC (17)	America	Lower diversity **Phyla:** Proteobacteria ↑ **Species:** A species in genus Blautia ↑, two species in genus Odoribacter and an unnamed genus (family Rikenellaceae) ↓		(31)
van der Meulen, et al. (2019)	SLE (30) vs. HC (965)	Netherlands	Lower bacterial richness **Phyla:** Firmicutes/Bacteroidetes ↓ **Species:** Bacteroides ↑		(51)
Azzouz, et al. (2019)	SLE (61) vs. HC (17)	America	Species richness diversity ↓ **Family:** Veillonellaceae ↑, Ruminococcaceae ↓ **Genus:** Ruminococcus ↑ **Species:** Ruminococcus gnavus (RG)↑, Bacteroides uniformis ↓	SLE patients with high disease activity showed significantly restricted microbiota diversity. RG relative abundance correlated with lupus disease activity. Anti-RG antibodies were directly correlated with SLEDAI scores and anti-DNA levels, but negatively correlated with C3 and C4. Patients with active lupus nephritis showed highest levels of serum anti-RG antibodies.	(58)
Li, et al. (2019)	SLE (40) vs. HC (22)	China	Lower richness **Phyla:** Firmicutes / Bacteroidetes ↓, Tenericutes, Mollicutes, and RF39 ↓ **Family:** Streptococcaceae and Lactobacillaceae ↑ **Genus:** Faecalibacterium ↓, Roseburia ↓, Streptococcus ↑, Lactobacillus ↑, Megasphaera ↑ **Species:** prausnitzii ↓, Streptococcus. Anginosus ↑, Lactobacillus. mucosae ↑	The genus Streptococcus was associated with the activity of SLE.	(59)
Zhang, et al. (2019)	SLE (92) vs. HC (217)	China	**Phyla:** Proteobacteria, Bacteroidetes and Actinobacteria ↑, Firmicutes ↓ **Family:** Lachnospiraceae, Ruminococcaceae and Veillonellaceae ↓, Bacteroidaceae, Streptococcaceae ↑ **Genus:** Ruminococcus, Bacteroides, Klebsiella,Erysipelotrichaceae ↑, Haemophilus, Faecalibacterium, Clostridium IV ↓	The proportion of Ruminococcus was correlated with the absolute counts of Tregs and the ratio of Th1/Th2 and Th17/Treg.	(66)
Guo, et al. (2020)	SLE-G (17) vs. SLE + G (20) + HC (20)	China	**Phyla:** Bacteroidetes ↑, Firmicutes/Bacteroidetes ↓ **Genus:** Gemmiger, Lactococcus, Bifidobacterium, Streptococcus, and Desulfovibrio ↓	SLE patients treated with glucocorticoid had a similar gut microbial community with healthy controls. Bacteroides, Succinivibrio, Bilophila, and Parabateroides were positively correlated with IL-17, TWEAK, IL-2R, IL-21, IL-35, IL-10, and IFN-γ. Dialister and Gemmiger were negatively correlated with immune factors IL-17, IL-2R, and IL-35.	(67)
Gerges, et al. (2021)	SLE (20) vs. HC (20)	Egypt	**Phyla:** Firmicutes ↓, Bacteroidetes ↑, Firmicutes/Bacteroidetes ↓ **Genus:** Lactobacillus ↓	Firmicutes/Bacteroidetes ratio was inversely correlated with SLEDAI-2K scores for disease activity.	(52)
Liu, et al. (2021)	SLE (35) vs. HC (35)	China	Reduced bacterial richness and diversity **Family:** Ruminococcaceae ↓ **Genus:** Lactobacillus, Prevotella and Blautia ↑, Bifidobacterium ↓	SLE patients with arthritis showed lack of Bifidobacterium. The abundance of Acholeplasma, Capnocytophaga and Leptotrichia were negatively correlated with SLEDAI score. The abundance of Akkermansia, Bacteroides, and Ruminococcus were negatively correlated with the serum levels of C3.	(60)
Wang, et al. (2022)	SLE (19) vs. HC (19)	China	**Phyla:** Acidobacteria, Gemmatimonadetes, Planctomycetes↓ **Genus:** Streptococcus, Veillonella, ClostridiumXI and Rothia ↑, Acidobacteria_Gp6, Croceibacter, Bacillariophyta, Acetatifactor, Helicobacter, Turicibacter, Butyricicoccus and Alloprevotella ↓	Patients with lupus nephritis showed a considerable increase in Streptococcus and a reduction in Turicibacter. Clostridium_XlVa, Lachnospiracea_incertae_sedis and Parasutterella OTUs mainly covaried with clinical features of SLE.	(48)
Toumi, et al. (2022)	SLE (16) vs. HC (76)	France	Decreased alpha-diversity **Phyla:** Firmicutes / Bacteroidetes↓, Tenericutes↓ **Family:** Tannerellaceae ↑ **Genus:** Alistipes, Flintibacter and Parabacteroides↑ **Species:** A. onderdonkii ↑	Active SLE patients had a significantly lower F/B ratio than inactive SLE group.	(37)

↑, higher abundance or enriched; ↓, lower abundance or depleted.

In recent years, studies have further confirmed the association between gut microbiota and SLE, and pointed out a causal relationship between the two. Xiang et al. (68) performed a meta-analysis including 11 case-control studies conducted in five countries and nine cities, and found increased abundance in *Enterobacteriaceae* and *Enterococcaceae* and decreased abundance in *Ruminococcaceae* in the gut microbiota of patients with SLE. This study added to evidence that dysbiosis of gut microbiota is present in lupus patients. Furthermore, a two-sample mendelian randomization study found that *Actinobacteria*, *Bacillales*, *Coprobacter* and *Lachnospira* were inversely correlated with the risk of SLE, and *Bacilli*, *Eggertella* and *Lactobacillales* might be the risk factors of SLE. More importantly, this study showed causal effects of gut microbiota on SLE (69).

## Potential mechanisms linking gut microbiota to SLE

### Impaired intestinal barrier and pathogen translocation

The intestinal epithelium not only separates the host from the external environment, but also acts as a first-line innate immune defense against the entry of foreign antigens. A normal gut barrier has size selectivity to prevent the translocation of viable organisms and several PAMPs, which can trigger the systemic inflammation (70). When the intestinal barrier is compromised, pathogens can translocate to systemic circulation and internal organs. It has been reported that the gut microbiota and leaky gut are related to autoimmune diseases including type 1 diabetes (71) and multiple sclerosis (72). Many studies as follow have revealed that intestinal barrier impairment and pathogen translocation also occurred in lupus mice and patients.

In lupus mice models, Mu et al. (30) found that endotoxin levels were significantly higher in the blood of MRL/lpr mice compared to MRL/MP controls. MRL/Lpr mice had significantly abundant FITC-dextran in the blood when gavaged with FITC-dextran compared to MRL/MP mice. Vieira et al. (73) reported that fluorescein isothiocyanate (FITC)– dextran was found in the systemic circulation of (NZW × BXSB)F1 hybrid mice after being taken orally. *E. gallinarum* was detected in the mesenteric veins, mesenteric lymph nodes (MLN), spleen, and liver of (NZW × BXSB)F1 hybrid mice. Both TLR7.1 Tg and C57Bl/6 mice treated with imiquimod showed impaired gut barrier, which was reflected by leakage of FITC-dextran into the systemic circulation. *L. reuteri* was found to translate into the mesenteric lymph nodes (MLN), spleen, and liver of TLR7.1 Tg mice (32). Toral et al. (74) observed significantly higher endotoxin levels in plasma in NZBWF1 mice compared with the control group.

In healthy people, an intact intestinal barrier can prevent luminal contents and systemic IgG from leaking into the gut lumen. Calcitonin is a recognized biomarker of intestinal barrier deficiency (58). In SLE patients, increased fecal albumin and calprotectin were found in the stool sample (73). Consistently, Azzouz et al. (58) also observed an increase in IgG and a higher level of calprotectin in the fecal sample of SLE patients. These results suggested impaired gut barrier function in SLE patients. *E. gallinarum* was detected in liver biopsy samples from both lupus patients and autoimmune hepatitis patients (73). Elevated serum soluble CD14 and α1-acid glycoprotein levels were found in lupus patients, which indicated translocation of the gut microbiota (58).

Gut-leakage is also associated with lupus disease progression. Thim-Uam et al. (75) used dextran sulfate solution (DSS) to induce gut-leakage in FcGRIIb-/- mice and found that DSS-induced gut-leakage induced high anti-dsDNA immunoglobulin in serum, enhanced lupus features including proteinuria and serum creatinine, caused the gut translocation of molecular components of gut pathogens, enhanced MLN apoptosis and induced spleen apoptosis in FcGRIIb-/- mice. Silverman et al. (76) colonized C57BL/6 mice with individual *Ruminococcus gnavus* (RG) strains from lupus patients, and found that lupus-derived RG strains enhanced intestinal permeability, elevated serum levels of zonulin which is a regulator of tight junction formation between cells forming the intestinal barrier, and translocated to mesenteric lymph nodes. The level of intestinal permeability induced by RG has a significant correlation with anti-native DNA autoantibodies and serum IgG anti RG cell-wall lipoglycan antibodies.

Interestingly, the “leakiness” of the gut epithelium could be reduced or even reversed, and the translation of gut bacteria could be suppressed. Antibiotics reduced intestinal leakage and suppressed microbial translocation (73, 77). Intramuscular vaccination against *E. gallinarum* prevented *E. gallinarum* translocation into internal organs (73). Resistant starch tightened the gut epithelial barrier and reduced the translocation of *L. reuteri* (32). *Lactobacillus* supplementation enhanced the barrier function of the intestinal epithelium (74). All-trans-retinoic acid treatment reversed pristane-induced leaky gut (33). Treatment with larazotide acetate, a specific molecular antagonist of zonulin, completely reversed gut permeability (76).

In summary, intestinal microbiota translocates to systemic circulation and internal organs since the intestinal barrier is compromised, increases exposure to autoantigens through inducing or enhancing apoptosis of cells such as splenocytes, and ultimately leads to increased deposition of immune complexes in organs then aggravates lupus. Interestingly, some interventions can reduce or even reverse the intestinal leakage to alleviate lupus.

### Molecular Mimicry

Molecular mimicry is another potential mechanism linking gut microbiota to SLE. Gut microbiota can mimic autoantigens through their proteins and metabolites (78). In hosts carrying high risk human leukocyte antigen (HLA) genes, continued colonization of them with bacteria expressing cross-reactive epitopes may continuously activate cross-reactive autoreactive T cells in the gut, especially in the case of impaired intestinal barrier (79). Patients with SLE produce autoantibodies against Ro60 which is an evolutionarily conserved RNA binding protein, and the antibody against Ro60 is the most common and earliest preclinical anti-nuclear antibody (80). Greiling et al. (80) found that bacteria expressing Ro60 orthologs was present in the skin, oral, and gut of both lupus patients and healthy controls. Sera from SLE patients with positive anti-Ro60 immunoprecipitated commensal Ro60 ribonucleoproteins, and Ro60-containing bacteria from skin and activated human Ro60 autoantigen-specific CD4 memory T cell clones. Furthermore, colonization of germ-free mice with *Bacteroides thetaiotaomicron* containing Ro60 ortholog caused T and B cell responses against human Ro60 and glomerular immune complex deposition (80).

Anti-β2GP1 antibody is also a diagnostic index for SLE. β2GP1 ortholog expressed by *E. gallinarum* was found to induce anti-β2GP1 antibody (73). Ruff et al. (81) found that β2GPI-reactive memory CD4^+^ memory T cell clones and APS-derived β2GPI autoantibody cross-reacted with mimotopes expressed by a gut commensal *Roseburia intestinalis*. Moreover, oral gavage of (NZW x BXSB)F1 mice with *Roseburia intestinalis* led to increased anti-human β2GPI IgG autoantibodies and thrombotic events.

In addition, Azzouz et al. (58) found that antigen in *Ruminococcus gnavus* strain CC55_001C could cross-react with anti-dsDNA antibodies. The peptide “YLYDGRIFI” similar to human Sm antigen epitope from *Odoribacter splanchnicus* increased secretion of IFN-γ and IL-17A. And the peptide “DGQFCM” mimicking human Fas antigen from *Akkermansia muciniphila* specifically binds to the IgG produced by memory B cells from lupus patients (27).

**Figure 2:**
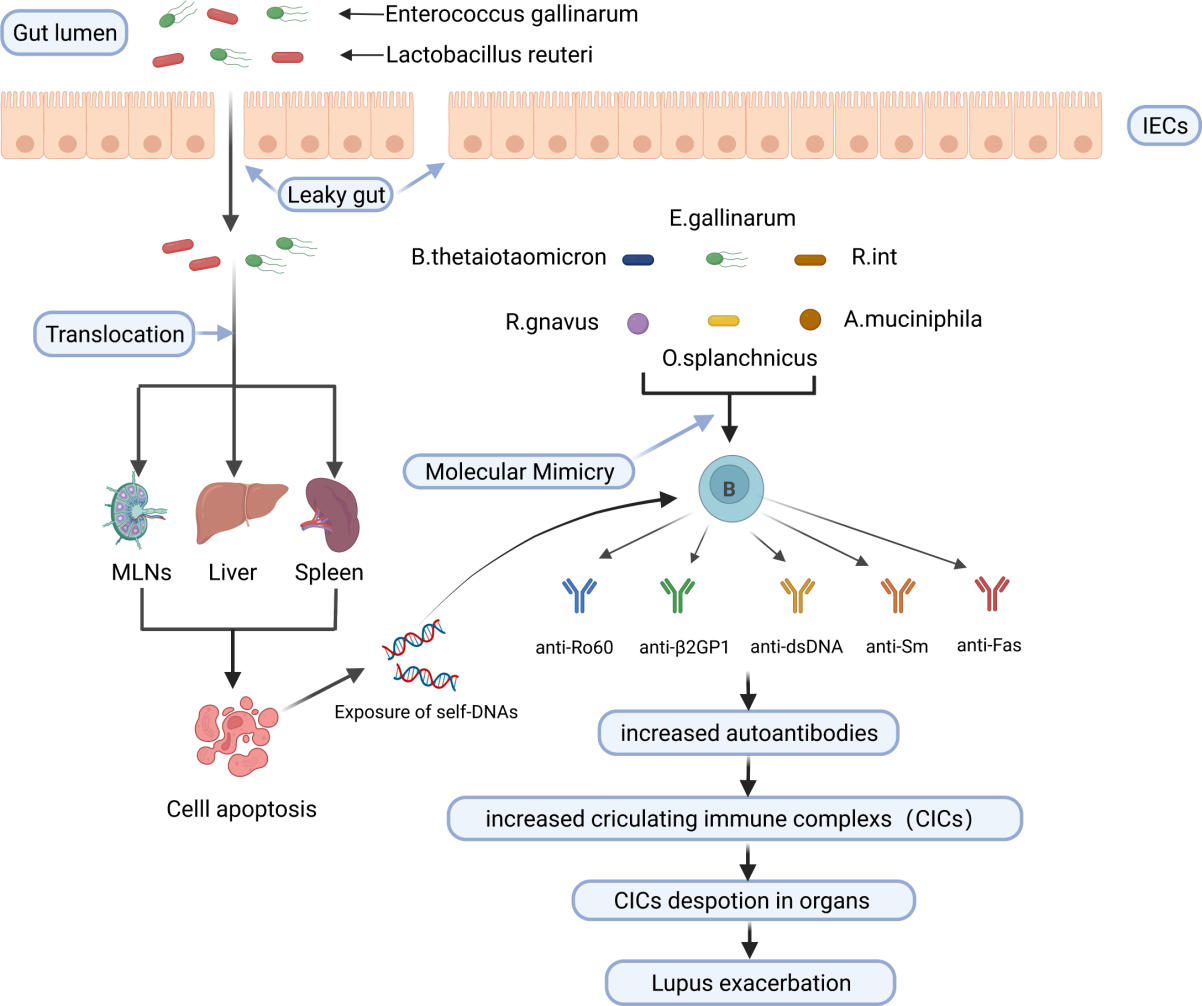
Potential mechanisms linking the gut microbiota to SLE. *E. gallinarum* and *L. reuteri* can translocate to internal tissues and organs such as mesenteric lymph nodes (MLN), liver, and spleen in the case of impaired gut barrier function, increase exposure to autoantigens through inducing or enhancing apoptosis of cells such as splenocytes, and increase the production of autoantibodies. *B. thetaiotaomicron*, *E. gallinarum*, *R. int*, *R. gnavus*, *O. splanchnicus* and *A. muciniphila* trigger cross-reactive B cell response through molecular mimicry of human autoantigens by their bacterial orthologs. Finally, increased circulating immune complexs deposit in organs and exacerbate lupus. IECs, intestinal epithelial cells; MLNs, mesenteric lymph nodes; *B. thetaiotaomicron*, *Bacteroides thetaiotaomicron*; *R. int*, *Roseburia intestinalis*; *R. gnavus*, *Ruminococcus gnavus; O. splanchnicus, Odoribacter splanchnicus; A. muciniphila, Akkermansia muciniphila.* Created by BioRender.com.

In conclusion, gut microbiota activates autoreactive T and B cells and triggers autoimmunity through encoding autoantigen orthologues and non-orthologous mimotopes of autoantigens, thus contributing to the development of SLE. Potential mechanisms linking the gut microbiota to SLE are shown in **Figure 2**.

## Immune dysregulation associated with gut microbiota in SLE

### Immune cells dysregulation

The imbalance between anti-inflammatory regulatory T(Treg) and inflammatory T-helper 17 (Th17) cells plays a significant role in the pathogenesis of SLE (82, 83). Recently, some studies have revealed that some specific pathogens contributed to Th17/Treg imbalance in lupus mice models. Vieira et al. (73) found that Th17 cells were induced in the small intestinal lamina propria and mesenteric lymph nodes of C57BL/6 mice monocolonized with *E. gallinarum*. The induction of Th17 and autoantibodies by *E. gallinarum* was eliminated by the administration of a selective AhR antagonist, which indicates that *E. gallinarum* promotes autoimmunity by AhR signaling. Zhang et al. (66) reported that the proportion of *Ruminococcus* was associated with the absolute counts of Treg cells and the ratio of Th17/Treg and Th1/Th2. NZM2410 mice colonized with segmented filamentous bacteria (SFB) showed increased Th17 cells in small intestinal lamina propria (40). Besides, the intestinal microbiota from SLE patients or mice also induced Th17/Treg imbalance in germ-free mice. Germ-free mice treated with feces from SLE mice showed increased B cells and significantly less abundant Treg cells mice in intestinal mucosa compared to those treated with feces from B6 mice (41). Ma et al. (43) found an increase in the frequency of Th17 cells and a reduction in Treg cells in the spleen of germ-free mice gavaged with feces from SLE patients. Additionally, the frequency of Tfh cells in circulation (84) and the Tfh/Tfr ratio (85) are positively correlated with lupus activity. Choi et al. (42) found that germ-free B6 mice that received feces from triple congenic lupus-prone mice exhibited an increased frequency of Tfh cells and a decreased ratio of Tfr to Tfh.

Interestingly, probiotic treatment could restore the balance between Treg cells and Th17 cells. *Bifidobacterium* can maintain the balance of Treg/Th17/Th1 by suppressing the excessive activation of CD4^+^ lymphocytes in SLE patients (86). *Lactobacillus* supplement could increase Treg cells and reduce Th17 cells to restore Th17/Treg balance (30, 74, 87). *Bacteroides fragilis* treatment restored the Th17/Treg balance and ameliorated the lupus activity of MRL/lpr mice (88). *Lactobacillus fermentum* CECT5716 and *Bifidobacterium* breve CECT7263 treatment restored the Th17/Treg balance in MLNs and reduced vascular Th1, and Th17 infiltration to restore endothelial function in a mouse lupus model induced by activating TLR-7 (89). Furthermore, Kim et al. (36) found that *Lactobacillus acidophilus* modulated Th17/Treg balance in MRL/lpr mice by the SIGNR3 pathway. In addition, regulated B (Breg) cells can exert immunosuppressive effects and support immune tolerance (90). Mu et al. (91) reported that oral administration of bacterial DNA could induce Breg cells and alleviate lupus.

### Cytokines dysregulation

In a series of immune-mediated diseases, cytokines act as critical mediators of inflammation and tissue damage (92). Many studies have revealed that T cells produced cytokines abnormally in SLE patients (93). IL-6, a pro-inflammatory cytokine produced by activated antigen-presenting cells and T cells, is known to promote B cells to produce antibodies (94) and suppress Treg cells (95). IL-17 is dysfunctional in SLE and promotes the disease progression (96). A meta-analysis displayed that the level of IL-17 has a positive correlation with lupus activity (97). IL-10 can inhibit kidney disease by inhibiting IFNγ-mediated production of IgG2a, which is a major immune deposit in the kidney of MRL/lpr mice (98). Mu et al. (30) found that *Lactobacillus* treatment decreased IL-6 and increased IL-10 production in the gut contributing to an anti-inflammatory environment. In SLE patients, twelve cytokines including IFN-γ, IL-1β, IL-2R, IL-6, IL-8, IL-10, IL-17, IL-21, IL-22, IL-35, TNF-α and TWEAK displayed higher expression levels compared to healthy controls. *Bacteroides*, *Bilophila*, *Parabateroides*, and *Succinivibrio* were positively correlated with IL-2R, IL-10, IL-17, IL-21, IL-35, TWEAK, and IFN-γ. *Dialister* and *Gemmiger* had a negative correlation with IL-2R, IL-17, and IL-35 (67). Yao et al. (61) also reported that SLE patients displayed higher serum levels of IL-2 and IL-6. The abundance of *Roseburia* and *Faecalibacterium* was inversely correlated with IL-6, the abundance of *Roseburia* had a negative correlation with IL-2, and the abundance of *Bacteroides* had a positive correlation with IL-2. Therefore, gut microbiota dysbiosis is associated with dysregulated cytokines in SLE. Interestingly, SLE patients treated with synbiotics had a significant decrease in serum IL-6 (54). Type I IFN, primarily produced by plasmacytoid dendritic cells, is a major pathogenic factor in SLE (99, 100). Vieira et al. (73) found that the presence of *E. gallinarum* upregulated Enpp3, which can increase the number of plasmacytoid dendritic cells (pDCs). Both murine hepatocytes and human hepatocytes induced type I interferon under the stimulation of *E. gallinarum*. Under specific-pathogen-free and gnotobiotic conditions, *L. reuteri* colonization increased plasmacytoid dendritic cells and interferon signaling (32).

**Figure 3:**
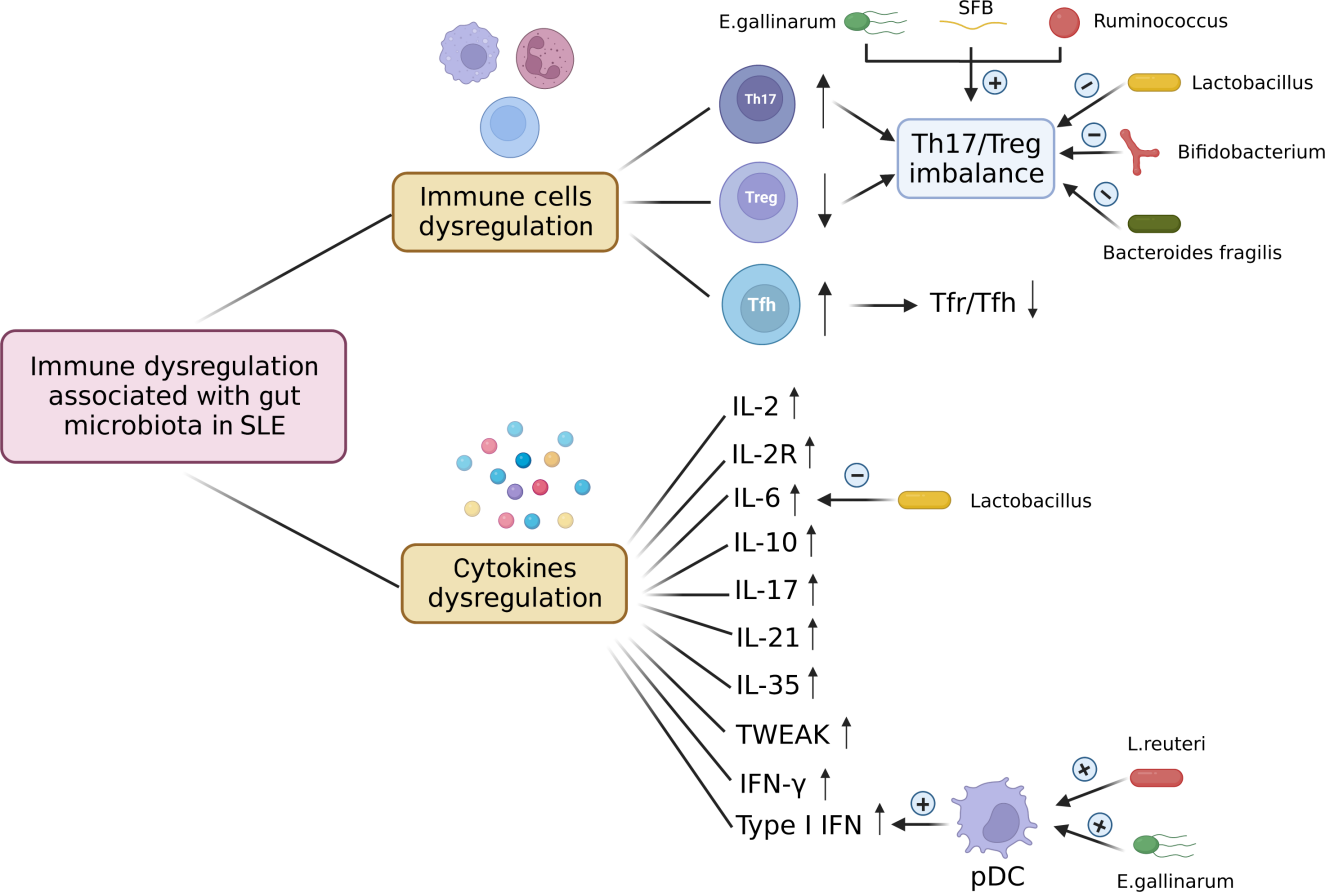
Immune dysregulation associated with gut microbiota in SLE. The immune dysregulation associated with gut microbiota in SLE occurs not only at the immune cell level but also at the cytokine level. Immune cells dysregulation includes increased Th17 cells, decreased Treg cells, and increased Tfh cells, which leads to the Th17/Treg imbalance and the decreased ratio of Tfr to Tfh. *E. gallinarum*, SFB, and *Ruminococcus* induce Th17/Treg imbalance by increasing Th17 cells and decreasing Treg cells. *Lactobacillus*, *Bifidobacterium* and *Bacteroides fragilis* can restore the Th17/Treg balance. The levels of cytokines associated with gut microbiota IL-2, IL-2R, IL-6, IL-10, IL-17, IL-21, IL-35, TWEAK, IFN-γ, and type I IFN are up-regulated in SLE. *E. gallinarum* and *L. reuteri* increase the number of plasmacytoid dendritic cells (pDCs) and promote the expression of type 1 interferon. *Lactobacillus* treatment can decrease the level of IL-6. pDC, plasmacytoid dendritic cell; SFB, segmented filamentous bacteria; *E. gallinarum*, *Enterococcus gallinarum*; *L. reuteri*, *Lactobacillus reuteri.* Created by BioRender.com.

To sum up, gut microbiota dysbiosis contributes to the immune dysregulation in SLE, the immune dysregulation associated with gut microbiota in SLE occurs not only at the immune cell level but also at the cytokine level. The immune dysregulation associated with gut microbiota in SLE is shown in **Figure 3**.

## Gut microbiota: potential diagnostic and therapeutic value for SLE

### Gut microbiota modulation to treat lupus mice models

*Lactobacillus*, known as probiotic, plays an anti-inflammatory role in autoimmune disease (101). Many studies have shown the beneficial effects of *Lactobacilli* supplementation in SLE mice. Hsu et al. (102) found that supplementary treatment of *Lactobacillus paracasei* GMNL-32, *Lactobacillus reuteri* GMNL-89 and *L. reuteri* GMNL-263 mitigated hepatic inflammation and apoptosis in NZB/W F1 lupus-prone mice. *Lactobacillus* treatment attenuated lupus nephritis through reducing renal deposition of IgG2a in MRL/lpr mice (30). NZBWF1 mice exhibited reductions in blood pressure, cardiac and renal hypertrophy, splenomegaly and lupus activity after *Lactobacillus fermentum* CECT5716 treatment (74). Manirarora et al. (87) reported that feeding *Lactobacilli* might delay lupus progression in BWF1 mice. *Lactobacillus acidophilus* improved gut dysbiosis, decreased the renal inflammation and enhanced the therapeutic effect of tacrolimus in MRL/lpr mice. However, Luo. et al. (31) found that the greater abundance of a group of *Lactobacilli* in NZB/W F1 mice might be linked to more severe disease. *Lactobacillus reuteri* can drive autoimmunity in TLR7-dependent mouse models of SLE (32). *Lactobacillus* have different effects on various lupus mice models which represent different genetic or environmental conditions, which may indicate that probiotic supplementation in the treatment of lupus should be individualized based on genetic and environmental factors.

Some other interventions such as dietary interventions, drug therapy, vaccination, helminth therapy and fecal microbiota transplantation have also been shown to modulate gut microbiota and exert beneficial effects in lupus mice. Zhang et al. (29) found that retinoic acid treatment restored downregulated *Lactobacilli* and improved lupus symptoms in lupus-prone mice. All-trans-retinoic acid reduced circulatory and renal deposition of autoantibodies and suppressed the expression of proinflammatory cytokines and chemokines in kidney in Balb/c mice treated with pristane (33). Resistant starch decreased the abundance of *Lactobacill. reuteri* and prevented the development of systemic autoimmunity in TLR7.1 Tg mice (32). Johnson et al. (103) treated (SWR×NZB) F1 (SNF1) mice with acidic pH water and neutral pH water respectively, and found that the composition of gut microbiota is significantly different between two groups of mice. Mice treated with acidic pH water developed nephritis at a slower pace than those treated with neutral pH water. Low dietary tryptophan has been reported to prevented autoimmune pathology in lupus-prone mice (42). He et al. (34) found that butyrate supplementation could ameliorate gut microbiota dysbiosis and reduce kidney damage in MRL/lpr mice. Mu et al. (77) found that antibiotics treatment reshaped the gut microbiota by reducing potentially harmful bacteria and enriching potentially beneficial bacteria and ameliorated systemic autoimmunity as well as kidney histopathology in MRL/lpr mice. In NZBWF1 mice, antibiotics was also found to change the composition of gut microbiota, suppress the elevation of blood pressure, and reduce renal injury and disease activity (35). Antioxidant N-acetylcysteine altered the composition of gut microbiota and alleviated autoimmunity in MRL/lpr mice (38). (NZW × BXSB) F1 mice showed restored intestinal barrier function and alleviated lupus after being inoculated with the *Enterococcus gallinarum* vaccine (73). It was reported that gastrointestinal helminth infection could modulate the gut microbiota (104). Olia et al. (105) found that infection with *Hymenolepis microstoma* inhibited NZBWF1 mice from developing lupus symptoms including production of autoantibody, proteinuria, glomerular histopathology, and splenomegaly. Wang et al. (106) transferred the gut microbiota of MRL/lpr mice treated with prednisone into the blank MRL/lpr mice, and found that prednisone-regulated gut microbiota alleviated lupus but didn’t show side effects as prednisone in MRL/lpr mice. And prednisone-regulated gut microbiota might alleviate lupus by retaining the abundance of *Lactobacillus* and decreasing *Ruminococcus* and *Alistipes*. Therefore, interventions which can modulate the dysbiosis of gut microbiota, such as dietary interventions, drug therapy, vaccination, helminth therapy and fecal microbiota transplantation, could be potential treatments for lupus patients.

### Gut microbiota modulation to treat SLE patients

Drugs can also affect the gut microbiota of lupus patients. Some studies have reported that the altered gut microbiota in lupus patients treated with some drugs becomes similar to the gut microbiota of normal individuals. Guo et al. (67) also found a lower *Firmicutes*/*Bacteroidetes* ratio in SLE patients, and the ratio increased in those having undergone glucocorticoid treatment. Besides, patients treated with glucocorticoids had similar gut microbial community to healthy controls, which means that changes in the gut microbiome might represent a return to homeostasis. Li et al. (107) reported that the gut microbiota of SLE patients treated with proton pump inhibitors (PPIs) showed increased alpha-diversity and the alpha-diversity became similar to healthy controls. PPIs use is related to the increased abundance of beneficial commensals and the decreased abundance of certain opportunistic pathogenic genera such as *Escherichia*.

Furthermore, several studies have also attempted to treat SLE patients by modulating gut microbiota. Huang et al. (28) conducted the first fecal microbiota transplantation (FMT) clinical trial in active SLE patients by oral encapsulated fecal microbiome from healthy donors, and found that FMT treatment significantly reduced the SLEDAI-2K score and the level of serum anti-dsDNA antibody. They also observed a marked increase in SCFAs-producing bacterial taxa and a decrease in inflammation-related bacterial taxa, with CD4^+^ memory/naïve ratio and levels of IL-6 decreasing in the peripheral blood and production of SCFAs increasing in the gut after FMT. It was reported that SCFAs played a beneficial immune regulatory role in SLE (108). Widhani et al. (54) found that the supplementation of synbiotics, a combination of prebiotics and probiotics, suppressed the increase of hs-CRP, reduced IL-6 expression, increased the *Firmicutes*/*Bacteroidetes* ratio, and improved SLE disease activity index 2K score in SLE patients.

**Figure 4:**
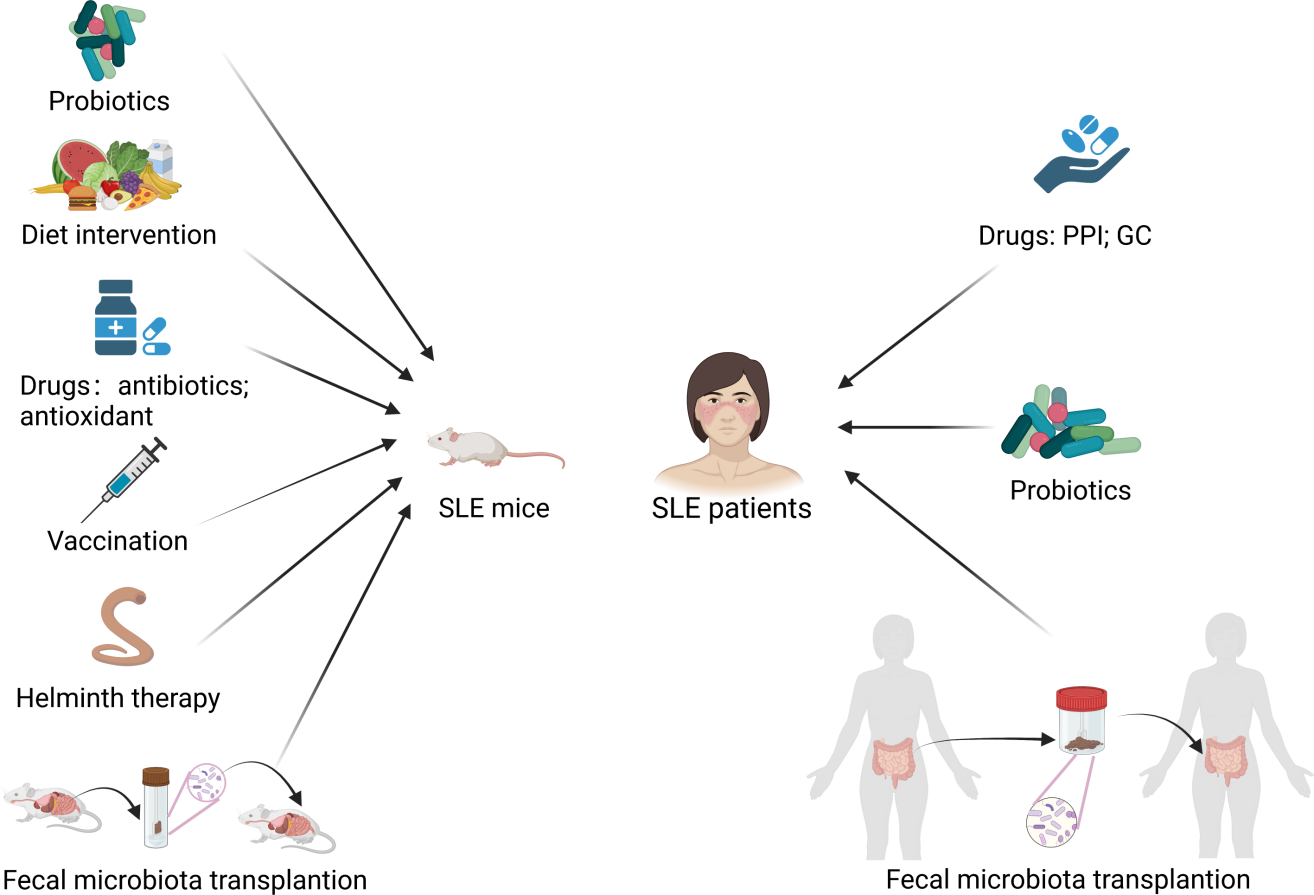
Modulation of gut microbiota treats SLE mice and patients. PPI, proton pump inhibitor; GC, glucocorticoid. Created by BioRender.com.

In addition, Li et al. (59) found that the random forest model could distinguish SLE patients from rheumatoid arthritis patients and healthy controls and predict the disease activity of SLE patients, which suggests the potential diagnostic value of gut microbiota as a potential biomarker. Therefore, the gut microbiota is not only a potential biomarker for the diagnosis and prognosis of SLE but also a potential target for the treatment of SLE. Interventions that attempt to treat SLE in mice models and humans by modulating the gut microbiota are summarized in **Figure 4**.

## Conclusion

The alteration in the gut microbiota of lupus mice and SLE patients is characterized by an increase in detrimental bacteria and a decrease in beneficial bacteria. Gut microbiota dysbiosis triggers autoimmunity through the potential mechanisms of translocation and molecular mimicry, leading to immune cells dysregulation (e.g., Th17/Treg imbalance) and cytokines dysregulation (e.g., increased expression of type I interferon), thereby contributing to the development and progression of SLE. Some interventions to modulate gut microbiota, such as dietary intervention, drug therapy (e.g., antibiotic and antioxidant N-acetylcysteine), probiotic supplementation, helminth therapy, vaccination and fecal microbiota transplantation, are potential treatments for SLE. Furthermore, gut microbiota is not only a potential therapeutic target for SLE but also a potential biomarker for the diagnosis and prognosis of SLE. However, there are few studies on the intervention of modulating gut microbiota such as fecal microbiome transplantation in the treatment of SLE patients. More studies are needed to verify the feasibility, safety and effectiveness of this approach.

## Author contributions

TBZ contributed to the conception and design of this review. KJY performed most of the overall work for this report and wrote the first manuscript. YNX, JLW, YDL and XTC reviewed and checked the article. TBZ modified and polished the article, and reviewed the article. All authors contributed to the article and approved the submitted version.

## Glossary

**Table:**

SLE	Systemic lupus erythematosus
Anti-dsDNA	Anti-double-stranded DNA
L. reuteri	Lactobacillus reuteri
TLR 7	Toll-Like Receptor 7
PH	Potential of hydrogen
F/B	Firmicutes/Bacteroidetes
SLEDAI	Systemic lupus erythematosus disease activity index
ESR	Erythrocyte sedimentation rate
CRP	C reactive protein
E. gallinarum	Enterococcus gallinarum
RG	Ruminococcus gnavus
PPIs	Proton pump inhibitors
FMT	Fecal microbiota transplantation
SCFAs	Short-chain fatty acids
Hs-CRP	Hypersensitive C-reactive protein
IL	Interleukin
MLN	Mesenteric lymph node
Ig	Immunoglobulin
CD14	Cluster of differentiation 14
HLA	Human leukocyte antigen
Tregs	Regulatory T cells
Th cells	T-helper cells
AhR	Aryl hydrocarbon receptor
SFB	Segmented filamentous bacteria
Tfh cells	T follicular helper cells
Tfr cells	Follicular regulatory T cells
Breg cells	Regulated B cells
IFN	Interferon
TNF	Tumor necrosis factor
TWEAK	TNF-like weak inducer of apoptosis
pDCs	Plasmacytoid dendritic cells
